# Exploring the Dynamics of Dietary Self-Monitoring Adherence Among Participants in a Digital Behavioral Weight Loss Program: Model Development Study

**DOI:** 10.2196/65431

**Published:** 2025-04-25

**Authors:** Hui Lin, Min Yang, Zhiheng Zhou, Yu Zhang, Ning Deng

**Affiliations:** 1 Pingshan Hospital Southern Medical University Shenzhen China; 2 Pingshan District Peoples' Hospital of Shenzhen Shenzhen China; 3 School of Public Health Zhejiang University School of Medicine Hangzhou China; 4 Center of Clinical Big Data and Analytics The Second Affiliated Hospital Zhejiang University School of Medicine Hangzhou China; 5 School of Biomedical Engineering Southern Medical University Guangzhou China; 6 Ministry of Education Key Laboratory of Biomedical Engineering College of Biomedical Engineering and Instrument Science Zhejiang University Hangzhou China

**Keywords:** self-monitoring of dietary behavior, ACT-R architecture, digital health interventions, adherence dynamics, goal pursuit, habit formation, tailored feedback interventions, weight loss program, computational behavioral science

## Abstract

**Background:**

Self-monitoring of dietary behaviors is typically a central component of behavioral weight loss programs, and it is widely recognized for its effectiveness in promoting healthy behavior changes and improving health outcomes. However, understanding the adherence dynamics of self-monitoring of dietary behaviors remains a challenge.

**Objective:**

We aimed to develop a prognostic model for adherence to self-monitoring of dietary behaviors using the Adaptive Control of Thought-Rational (ACT-R) cognitive architecture and to qualitatively investigate adherence dynamics and the impact of various interventions through model-based analyses.

**Methods:**

The modeling data were derived from a digital behavioral weight loss program targeting adults who expressed a willingness to improve their lifestyle. Participants were assigned to 1 of 3 intervention groups: self-management, tailored feedback, and intensive support. ACT-R, a cognitive architecture simulating human cognitive processes, was used to model adherence to self-monitoring of dietary behaviors over 21 days, focusing on the mechanisms of goal pursuit and habit formation. Predictor and outcome variables were defined as adjacent elements in the sequence of self-monitoring of dietary behaviors. Model performance was evaluated using mean square error, root mean square error (RMSE), and goodness of fit. Mechanistic contributions were visualized to analyze adherence patterns and the impacts of different interventions.

**Results:**

The total sample size for modeling was 97, with 49 in the self-management group, 23 in the tailored feedback group, and 25 in the intensive support group. The ACT-R model effectively captured the adherence trends of self-monitoring of dietary behaviors, with RMSE values of 0.099 for the self-management group, 0.084 for the tailored feedback group, and 0.091 for the intensive support group. The visualized results revealed that, across all groups, the goal pursuit mechanism remained dominant throughout the intervention, whereas the influence of the habit formation mechanism diminished in the later stages. Notably, the presence of tailored feedback and the higher levels of social support were associated with greater goal pursuit and more sustained behavioral practice.

**Conclusions:**

This study highlights the potential of ACT-R modeling for dynamic analysis of self-monitoring behaviors in digital interventions. The findings indicate that tailored feedback combined with intensive support may significantly improve adherence. Future studies should (1) extend the intervention duration to explore sustained adherence mechanisms, (2) integrate social cognitive factors to capture behavioral compliance insights, and (3) adapt dynamic models to inform just-in-time adaptive interventions for broader applications.

## Introduction

### Overweight and Obesity Trends and Importance of Self-Regulation

According to a World Health Organization report, in 2022, 43% of adults globally were classified as overweight and 16% were classified as obese [1]. The prevalence of obesity has more than doubled worldwide from 1990 to 2022 [2,3]. Overweight and obesity are not only deleterious to individual health, significantly elevating the risk of diabetes and cardiovascular diseases, but also impose a substantial economic burden on global health care systems [4-6].

Behavioral weight loss programs are critical interventions in the escalating obesity epidemic [7,8]. Empirical evidence suggests that these programs, which incorporate self-regulation strategies, are significantly effective in facilitating weight loss [9]. Since the root cause of overweight and obesity lies in an imbalance between caloric intake and expenditure, self-monitoring of dietary behaviors is often considered as the cornerstone of behavioral weight loss programs [9]. Empirical studies have consistently demonstrated a positive correlation between adherence to self-monitoring of dietary behaviors and enhancements in both health behaviors and physiological outcomes. For instance, Teasdale et al [10] conducted a meta-analysis revealing that rigorous self-monitoring of diet behaviors significantly ameliorates dietary habits. Yu et al [11] established a method for self-monitoring of diet and vital signs, which showed significant improvements in healthy habit formation and disease prevention.

Despite the efficacy of self-monitoring of dietary behaviors, participant adherence tends to wane over time due to the labor-intensive nature of the approach and the absence of an efficient passive recording method [12,13]. Consequently, it is imperative to develop innovative strategies to bolster participation in self-monitoring of dietary behaviors [9].

### Strategies to Improve Adherence to Self-Monitoring of Dietary Behaviors

Potentially effective strategies to enhance adherence to self-monitoring of dietary behaviors are threefold. First, leveraging digital technologies can significantly expand the accessibility and convenience of self-monitoring. A systematic review demonstrated that adherence to self-monitoring supported by digital technologies was superior to traditional paper-based methods [14]. Second, applying other self-regulation strategies, such as tailored nutritional feedback, has been shown to be beneficial. Social cognitive theory emphasizes the importance of feedback in achieving successful behavior change [15]. It allows participants to compare their dietary behaviors with healthy dietary standards, obtaining useful information directly related to themselves [16-18]. This not only enhances participants’ intentions to adhere to self-monitoring of dietary behaviors but also increases their awareness and understanding of healthy dietary practices [19,20]. Third, providing emotional social support can enhance the engagement of individuals. Emotional social support, characterized by emotional communication, care, and understanding during social interactions, has been shown to mitigate the effects of self-regulatory depletion and sustain effective self-regulation [21,22]. Forming support groups among participants can enhance weight loss success and aid in maintaining a healthy lifestyle over the long term [23]. In summary, technical assistance, tailored feedback, and emotional social support within behavioral weight loss programs can improve adherence to self-monitoring of dietary behaviors, thereby increasing the likelihood of achieving successful and sustainable weight control.

Digital technologies, such as mobile apps, have provided enormous opportunities for obtaining continuous and fine-grained user behavior data. These data provide profound insights into individual trends and variations across different scales [24,25]. When combined with finely-tuned behavioral dynamic modeling methods, they offer unparalleled prospects for understanding behavior change under various intervention strategies [26-28].

Cognitive behavioral modeling based on neurocognitive architectures is considered foundational for predicting and explaining human behavior changes under behavioral interventions. Neurocognitive architectures, which are computational implementations of unified theories of cognition [29], have been established through mechanisms and constraints extensively studied and refined by psychological scientists [30]. These architectures offer a well-established framework for multi-timescale and multi-module explanations. Therefore, cognitive behavioral modeling based on neurocognitive architectures can dynamically distill high-level constructs from social cognitive and other behavior change theories [30].

### Introduction of the Adaptive Control of Thought-Rational Architecture

Adaptive Control of Thought-Rational (ACT-R) is a neurocognitive architecture that integrates physical, neurophysiological, behavioral, and cognitive mechanisms into a computational model [31]. It is a hybrid cognitive architecture consisting of 2 parts: the symbolic system and the subsymbolic system.

The symbolic system comprises various modules, with the central procedural module being the most crucial. It integrates all other modules into a whole. Each module corresponds to a specific brain region and interacts with associated buffers to retrieve and store information [31,32]. The model posits 2 types of memory: chunks and production rules. Chunks reside in the declarative module and have an “activation” attribute influenced by retrieval time, frequency, and recentness of memory. The most activated chunk is prioritized for retrieval into the buffer. Production rules, located in the procedural module, consist of conditional statements (“if”) and corresponding actions (“then”) and are characterized by a “utility” attribute. Rules that match conditions in the buffer and have the highest utility are most likely to be executed [31].

The subsymbolic system manages operations within the modules through a series of computational processes, including activation, retrieval, learning, and selection (Table 1) [31,33]. These processes elucidate how different types of memory are retrieved, stored, and updated during cognitive operations.

**Table 1:** Computational processes in the subsymbolic system of the Adaptive Control of Thought-Rational architecture.

Mechanism	Description	Equation	Parameter
Activation	Activation refers to the calculation of the activation level of a chunk, which comprises base-level activation and spreading activation. The former reflects the frequency with which a chunk is accessed, while the latter reflects the relationship between the chunk and its context.	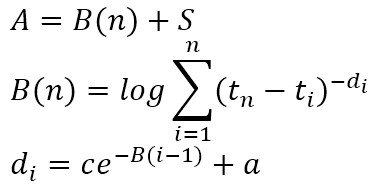	*A*: activation level of a chunk; *B*: base-level activation of a chunk; *S*: spreading activation of a chunk; *t*_*i*_: the time since the chunk’s *i*th access, *i*=1, …, n; *d*: decay rate
Retrieval	Retrieval refers to selecting and activating specific knowledge chunks from the declarative memory module. The higher the activation level of a knowledge chunk, the greater the probability of retrieval.	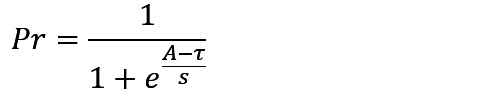	*Pr*: probability of retrieval of a chunk; *τ*: retrieval threshold of a chunk; *s*: activation noise
Learning	Learning refers to the calculation of the utility of production rules, where the repeated execution of rules accumulates rewards to form their utility.	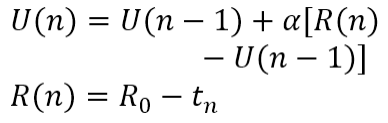	*U*: utility of a production rule; *α*: learning rate; *R*: reward for the execution of a production rule; *R*_*0*_: initial reward
Selection	Selection refers to the system’s process of choosing which production rule to execute based on their utility values.	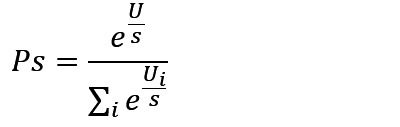	*Ps*: probability of selection of a production rule

In recent years, ACT-R has gradually become an important method for analyzing behavioral and cognitive dynamics in the field of public health. Pirroli [30] used ACT-R to model user behavioral data from a 28-day mHealth project called Dstress, examining the dynamic effects of social cognitive factors on exercise adherence [30]. The author then developed a computational model from goal striving to habit formation and studied the impact of different reminder schedules on health behavior change [33,34]. One study used the theory of planned behavior as a theoretical foundation for ACT-R modeling, analyzing the influences of several social cognitive structures on mask-wearing during the COVID-19 pandemic [35]. The success of these studies demonstrates that the fine-grained dynamic relationships between behavioral interventions and outcomes can be effectively quantified using ACT-R models. However, current analyses of adherence to self-monitoring of dietary behaviors are mostly descriptive and cross-sectional [9,36], with few dynamic analysis studies, and no ACT-R modeling studies have yet focused on adherence to self-monitoring of dietary behaviors.

### Health Diary for Lifestyle Change Program

This study is part of a digital behavioral weight loss program known as Health Diary for Lifestyle Change (HDLC). The program concentrates on strategies for self-monitoring, social support, and personalized feedback enabled by digital technology to facilitate healthy lifestyle formation and weight loss. Specifically, it includes (1) the design and continuous optimization of the implementation of these strategies, (2) the iterative development of a weight management system for delivering the intervention, and (3) a multi-stage evaluation of the program’s cost and effect.

The program has been piloted in diverse settings, including university campuses, internet-based enterprises, and residential communities, with ongoing studies exploring its broader application. Early research, such as a study targeting internet workers, used the RE-AIM (Reach, Effectiveness, Adoption, Implementation, and Maintenance) framework to evaluate intervention design, system functionality, implementation details, and weight loss outcomes [37]. This study focuses on data from a pilot implementation conducted among graduate students and university staff. Although the health education content has been tailored to meet the specific needs of different populations, the key interventions and system functionalities have remained consistent across all pilot studies, whether completed or ongoing.

While participants in the HDLC program are predominantly urban residents, disparities in health resource access persist between urban and rural areas [38,39]. However, with 1.09 billion internet users in China [40], programs like HDLC show potential to bridge these gaps by delivering adaptable and accessible health resources. The model developed in this study aims to inform cost-effective and automated strategies for enhancing the reach and efficiency of digital health interventions.

### Summary of Study Aims

Drawing from the seminal work of Pirolli et al [30,33,34], this study aims to achieve 2 primary objectives. First, it seeks to develop a model to predict the dynamics of adherence to self-monitoring of dietary behaviors among participants in the HDLC program using the ACT-R cognitive architecture, while evaluating the predictive performance of the resulting model. Second, it aims to conduct a qualitative analysis of the modeling outcomes across different intervention groups, with an emphasis on elucidating the influence of tailored feedback and social support strategies on enhancing participants’ adherence to self-monitoring of dietary behaviors. This study is reported in accordance with the TRIPOD (Transparent Reporting of a Multivariable Prediction Model for Individual Prognosis or Diagnosis) guidelines [41] and its updated version [42]. The checklist is provided in Multimedia Appendix 1.

## Methods

### Study Design

**Figure 1:**
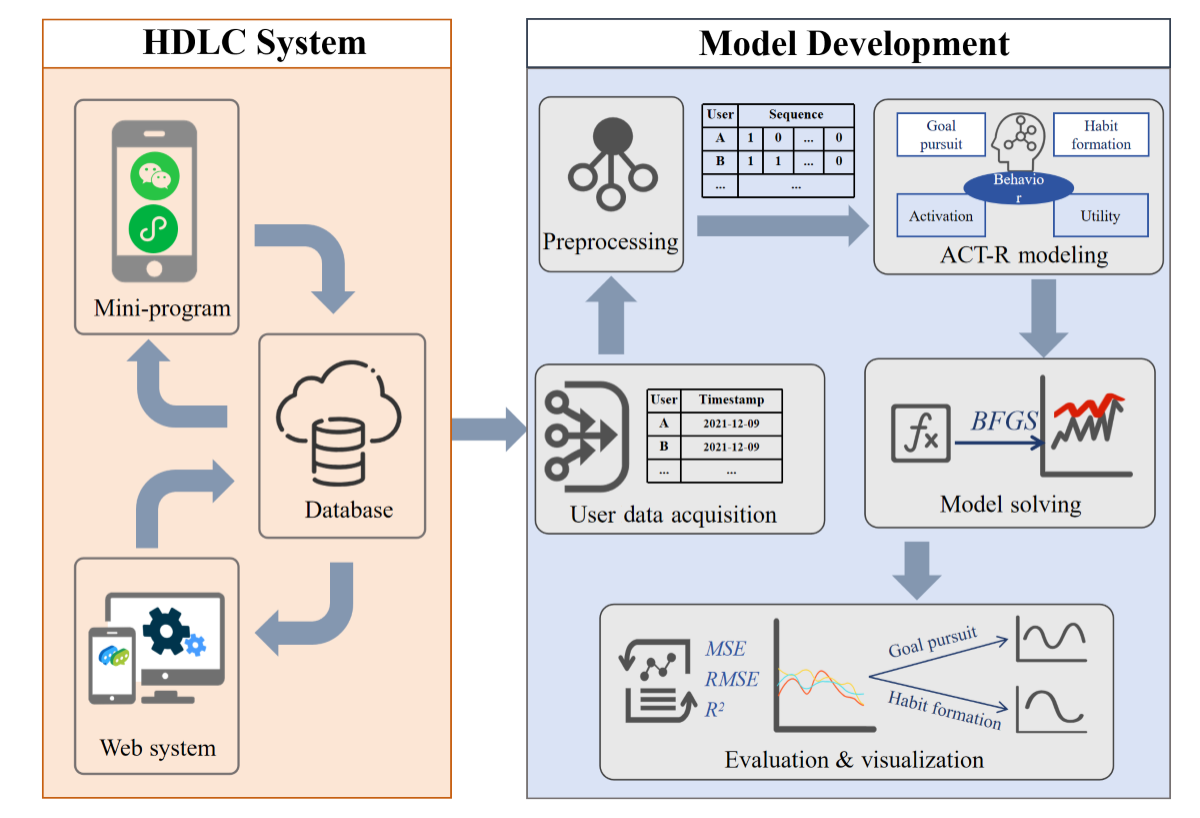
Methodological framework for Adaptive Control of Thought-Rational (ACT-R) model development. BFGS: Broyden-Fletcher-Goldfarb-Shanno algorithm; HDLC: Health Diary for Lifestyle Change program; MSE: mean square error; RMSE: root mean square error.

The methodology of this study presents the model development process from 2 perspectives (Figure 1). The first perspective provides an overview of the HDLC program, which served as the source of samples and data. The second perspective focuses on the construction, analysis, and evaluation of the ACT-R model, which is grounded in the mechanisms of goal pursuit and habit formation.

### Experiments

#### Participants and Allocations

Participants in the study were faculty, staff, and students from Zhejiang University in China. Recruitment announcements were posted on the university’s internal online forum, and interested individuals completed an online questionnaire to gather basic information such as gender, age, height, and weight. The recruitment began in November 2021 and lasted for 4 weeks, followed by a 28-day formal intervention. The study included adults who owned a smartphone and intended to adopt a healthy lifestyle. By including participants who explicitly expressed a willingness to adopt a healthy lifestyle, the program establishes a shared baseline of motivation to change. This psychological readiness, as a proximal determinant of behavioral adherence [43,44], allows for a more controlled evaluation of participants’ adherence to intended actions.

The HDLC program followed a quasiexperimental design based on natural allocation rather than randomization. Enrolled participants were initially assigned to groups based on BMI. Specifically, individuals with a BMI of 24 kg/m^2^ or higher were invited to join the tailored feedback group, and they visited a nutrition counseling center for a basic physical examination conducted by an onsite dietitian. Tailored feedback group participants were encouraged to form their own teams to engage in the intervention, thus creating the sample for the intensive support group. Participants with a BMI below 24 kg/m^2^ or those not selecting the tailored feedback intervention were assigned to the self-management group. The BMI cutoff point was defined based on authoritative reports and guidelines from China [45,46]. The 3 groups correspond to varying levels of social support: the intensive support group had the highest level, followed by the tailored feedback group and finally the self-management group. This allocation strategy was informed by practical and ethical considerations, as the national guidelines mention that individuals with higher BMI values may require more intensive support to achieve desired health outcomes.

#### HDLC Interventions and Systems

The HDLC program consisted of 2 phases: a 7-day preparation phase followed by a 21-day intervention. The entire intervention was delivered through 3 platforms: WeChat, a built-in mini-program, and a web system for user management by online dietitians.

WeChat, the largest instant messaging app in China with over 1 billion monthly users [47], offers diverse functions, including messaging, real-time information sharing, and built-in mini-programs. In the HDLC program, WeChat served as an instant communication channel for participants and health professionals, facilitating the delivery of key interventions such as reminders, feedback, and social support. Multimedia Appendix 2 provides details on the intervention. The mini-program, a lightweight app embedded in the WeChat platform, provided three core functionalities: (1) self-monitoring: enabling the recording and historical viewing of diet, exercise, and weight, with dietary records uploadable as photos; (2) tailored feedback: integrated with WeChat’s notification feature and allowing participants to receive nutritional feedback from dietitians based on their dietary records; and (3) health education: presenting educational content related to health in text, image, and video formats.

The web system enables online dietitians to view and manage user information and to edit nutritional feedback for dietary content according to the guidelines for diagnosis and treatment of obesity in China [45] and the Chinese guidelines for medical nutritional treatment of overweight/obesity [46]. To enhance efficiency, dietitians can use customized feedback templates. Both the mini-program and the web system were developed by Zhejiang University’s Institute of Medical and Health Information Engineering.

### Ethical Considerations

This study represents a secondary analysis of the HDLC program, which obtained ethical approval from the ethics committee of Zhejiang University’s School of Public Health in 2021 (approval number: ZGL202112-2) and was subsequently registered (registration number: ChiCTR2200055548). The ethics committee specifically reviewed and approved the secondary analysis plan to ensure compliance with ethical standards. During recruitment for the HDLC program, informed consent was obtained from all participants, explicitly allowing for secondary analyses of collected data, including system backend logs. To protect participant privacy, all data were anonymized prior to analysis through the removal of personally identifiable information and the replacement of unique identifiers with pseudonyms. Participants were incentivized for achieving weight loss through standardized rewards based on their progress. However, no additional compensation was provided specifically for adherence to self-monitoring of dietary behaviors.

### Measurements and Data Source

Baseline participant characteristics included gender, age, height (m), weight (kg), BMI (kg/m^2^), waist circumference (cm), body fat percentage, and labor intensity. Demographic characteristics (gender and age) and labor intensity were self-reported through an online recruitment questionnaire. Labor intensity was assessed using a single question: “Please classify your daily physical labor intensity according to the following categories: light (minimal effort, eg, sitting and walking), moderate (moderate effort, eg, brisk walking and light lifting), or heavy (substantial effort, eg, heavy lifting and vigorous exercise)” [48]. Subsequent in-person baseline assessments were conducted by a certified clinical nutritionist at the university nutrition counseling center. Height and weight were measured barefoot using an automated stadiometer system (SH-V5; Zhengzhou Shanghe Electronic Technology Co, Ltd). Waist circumference was measured at the midpoint between the iliac crest and the lowest rib using a constant-tension tape. Body fat percentage was measured using bioelectrical impedance analysis (InBody 270; Biospace China Co, Ltd) after an overnight fast (≥10 hours) upon waking. BMI was calculated from the measured height and weight. Follow-up evaluations replicated the baseline protocol to ensure methodological consistency.

The study defined the rate of adherence to self-monitoring of dietary behaviors, an overall measure of participant compliance, as the daily proportion of participants executing self-monitoring of dietary behaviors. Although not used as a modeling indicator, this study recognizes the value of weight-related outcomes in elucidating the broader context of study. Therefore, the reporting of changes in weight, BMI, waist circumference, and body fat percentage served as a supplement to the research background.

As an auto-regressive model, the ACT-R model predicted the probability of successful execution of self-monitoring of dietary behaviors on the next day based on its status on the current day. The study defined the sequence of adherence to self-monitoring of dietary behaviors over a continuous 21-day period as the modeling dataset. If a participant records at least two dietary entries in a single day [49], the sequence value for that day is assigned as 1; otherwise, it is assigned as 0. The source data, which consist of the exact times when users uploaded their dietary data, were extracted from the system backend logs. The data collection was conducted by a researcher who was blinded to the model’s outcomes and predictors at the end of the intervention.

According to the TRIPOD statements, the sample size in model development depends on the total number of outcome events [41]. For this study, the total number of outcome events was calculated by multiplying the length of the sequence of self-monitoring of dietary behaviors per participant by the number of participants. To ensure robustness, all available data were used to maximize the model’s performance and representation of diverse scenarios. However, a formal sample size calculation was not conducted prior to analysis. This is primarily due to the fact that ACT-R modeling currently lacks a widely accepted method for estimating the minimum required sample size. The absence of a formal sample size calculation has been acknowledged as a limitation and has been further discussed in the limitations section.

### Modeling Based on ACT-R

To model the impact of tailored feedback and social support on adherence to self-monitoring of dietary behaviors using ACT-R, it is essential to elucidate how ACT-R computes successful execution of self-monitoring of dietary behaviors and to articulate the structural representation of tailored feedback and social support strategies within the ACT-R architecture. Essentially, the successful execution of self-monitoring of dietary behaviors can be posited to involve 2 mechanisms.

First, the goal pursuit mechanism involves individuals consciously endeavoring to execute self-monitoring of dietary behaviors successfully, contingent upon the probability *P_G_* of retrieving the self-monitoring of dietary behaviors goal chunk and the probability *P_S_* of successful execution. *P_G_* is associated with the activation level of the self-monitoring of dietary behaviors goal chunk, while *P_S_* represents the goal pursuit success probability calculated based on ACT-R rule selection, taking into account the degree of intention-action translation.

Second, the habit formation mechanism suggests that the repeated practice of self-monitoring of dietary behaviors leads individuals to execute it automatically with a probability *P_H_*, even without explicit recall of the goals of self-monitoring of dietary behaviors. *P_H_* is related to the utility *U_SMDB_* of the self-monitoring of dietary behaviors production rule and represents the probability of successful habitual execution.

Since tailored feedback is only triggered after the execution of self-monitoring of dietary behaviors, this study hypothesizes that the influence of tailored feedback on the intention of self-monitoring of dietary behaviors is reflected in the coefficient of base-level activation. Furthermore, it is anticipated that varying intensities of social support will affect both the goal pursuit process and the habit formation process.

In summary, the model of self-monitoring of dietary behaviors formation and its influencing factors was constructed based on the 4 computational processes in the subsymbolic system of the ACT-R architecture, as outlined in Table 1 and detailed in Table 2.

**Table 2:** Computational processes of the behavior change mechanisms.

Mechanism and formula		Parameter description
**Output**		
	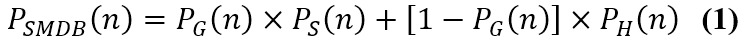	Successful probability of self-monitoring of dietary behaviors execution
**Goal pursuit**		
	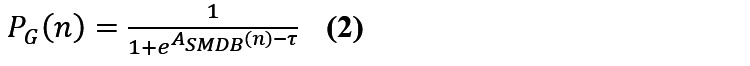	Retrieval probability of the self-monitoring of dietary behaviors goal chunk
	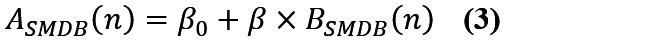	Total activation level of the self-monitoring of dietary behaviors chunk
	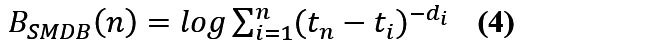	Base-level activation of self-monitoring of dietary behaviors
	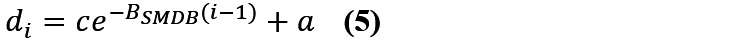	Decay coefficient of the base-level activation
	τ	Activation threshold for retrieval
	β_0_	Intercept term of the total activation
	β	Coefficient term of the total activation representing the influence of tailored feedback
	c, a	Control parameter of the decay coefficient
	t_i_	Time interval between the *i*th execution of self-monitoring of dietary behaviors and the first execution *i*=1, 2, ..., n
	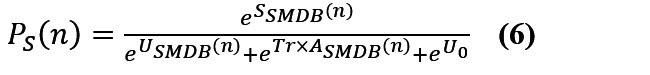	Probability of successful self-monitoring of dietary behaviors execution based on the goal pursuit mechanism
	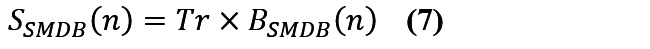	Activation level of the self-monitoring of dietary behaviors chunk after intention-behavior translation
	Tr	Intention-action transformation coefficient
**Habit formation**		
	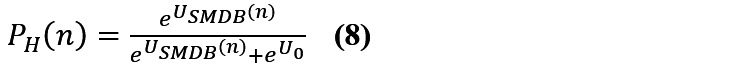	Probability of successful self-monitoring of dietary behaviors execution based on the habit formation mechanism
	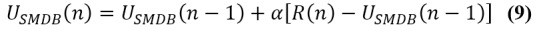	Utility of the self-monitoring of dietary behaviors production rule
	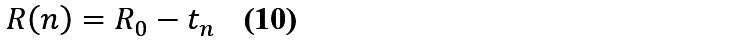	Reward for executing the self-monitoring of dietary behaviors production rule
	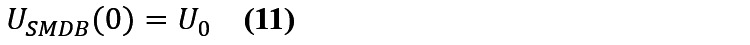	Initial utility
	α	Learning rate of the self-monitoring of dietary behaviors production rule
	R_0_	Initial reward

### Statistical Analysis

Descriptive analysis was conducted on participant characteristics. Anthropometric outcomes were analyzed as secondary endpoints using complete case analysis. Participants missing any postintervention measurements were excluded from these supplemental analyses but retained in the primary adherence modeling. Intergroup differences for continuous variables were assessed using either ANOVA or the Kruskal-Wallis test (if not normally distributed). Categorical variables were analyzed using the chi-square test. Changes in outcomes within groups were analyzed using paired *t*-tests. The Kruskal-Wallis test was used to analyze differences in the rates of adherence to self-monitoring of dietary behaviors among the 3 groups, and then, the Dunn test was used for multiple comparisons to explore specific intergroup differences.

Modeling data preprocessing excluded participants with consecutive 21-day zero adherence records, as these sequences lacked the temporal variance required for behavioral dynamics modeling. The values for the cognitive architecture’s system parameters (c, a, s, and α) were adopted from a study that used ACT-R to explore the impact of self-efficacy scores and different reminder strategies on health behavior changes [33]. The model included 5 free parameters: the coefficient *β* and intercept *β_0_* of the total activation, the intention-action transformation coefficient *Tr*, and the initial reward *R_0_* and utility *U_0_* of the production rules.

The study solved a set of model coefficients for each intervention group. The quasi-Newton method, specifically the Broyden-Fletcher-Goldfarb-Shanno (BFGS) algorithm, was employed to fit the trend curves of the rates of adherence to self-monitoring of dietary behaviors over 21 consecutive days for the 3 groups. After setting initial parameter values, the adherence sequences of individual participants in each group were input into the above-mentioned model to calculate the probability of successful execution of self-monitoring of dietary behaviors (*P_SMDB_*). The objective function aimed to minimize the difference between the proportion of participants adhering to self-monitoring of dietary behaviors and the model output. After fitting, the model was evaluated by calculating the mean square error (MSE), root mean square error (RMSE), and goodness of fit (*R*^2^). A bootstrapping method was used to estimate the 90% CI for the MSE and RMSE by resampling the dataset to generate 1000 values, from which the interval was derived. The Ljung-Box test was subsequently conducted to analyze the auto-correlation of residuals. Finally, the study qualitatively analyzed and visualized the contributions of the 2 mechanisms to outcomes and the impact of various interventions on adherence to self-monitoring of dietary behaviors.

## Results

### Descriptive Analyses

A total of 115 participants enrolled online for the study, and they had an average age of 22.12 years (Table 3). Among them, 80.8% (93/115) were female and 88.7% (102/115) reported light labor intensity. There were no significant differences among the 3 groups regarding gender (*χ*^2^_2_=3.09; *P*=.21) or labor intensity (*χ*^2^_2_=2.77; *P*=.25). Multiple comparison analyses indicated no significant age differences between the groups (self-management vs tailored feedback: *P*=.10; self-management vs intensive support: *P*=.19; tailored feedback vs intensive support: *P*>.99).

**Table 3:** Baseline characteristics of the participants in the study groups.

Characteristic		Self-management group (n=66)	Tailored feedback group (n=24)	Intensive support group (n=25)
Age (years), mean (SD)		24.94 (3.24)	28.71 (7.68)	26.77 (4.07)
Gender (female), n (%)		57 (86)	18 (75)	18 (72)
**Labor intensity, n (%)**				
	Light	60 (91)	19 (79)	23 (92)
	Medium or heavy	6 (9)	5 (21)	2 (8)
Height (cm), mean (SD)		163.72 (7.12)	166.38 (8.57)	163.92 (9.20)
Weight (kg), mean (SD)		57.06 (7.98)	73.58 (16.43)	67.88 (12.20)
BMI (kg/m^2^), mean (SD)		21.16 (1.91)	26.42 (4.76)	25.21 (3.60)

The mean rates of adherence to self-monitoring of dietary behaviors were 0.55 (SD 0.38) in the self-management group, 0.72 (SD 0.29) in the tailored feedback group, and 0.83 (SD 0.20) in the intensive support group. The Kruskal-Wallis test indicated significant differences in the mean rates of adherence to self-monitoring of dietary behaviors among the 3 groups (*P*=.04). Post-hoc comparisons revealed that the intensive support group had a significantly higher adherence rate than the self-management group (*P*=.04). Detailed information regarding weight-related outcomes is provided in Multimedia Appendix 3.

### Model Specification and Performance

The final modeling sample included 49 participants in the self-management group (74% retention), 23 in the tailored feedback group (96% retention), and 25 in the intensive support group (100% retention). Given that the adherence period was 21 days, the self-management, tailored feedback, and intensive support groups recorded 1029, 483, and 525 instances of the outcome event, respectively.

**Figure 2:**
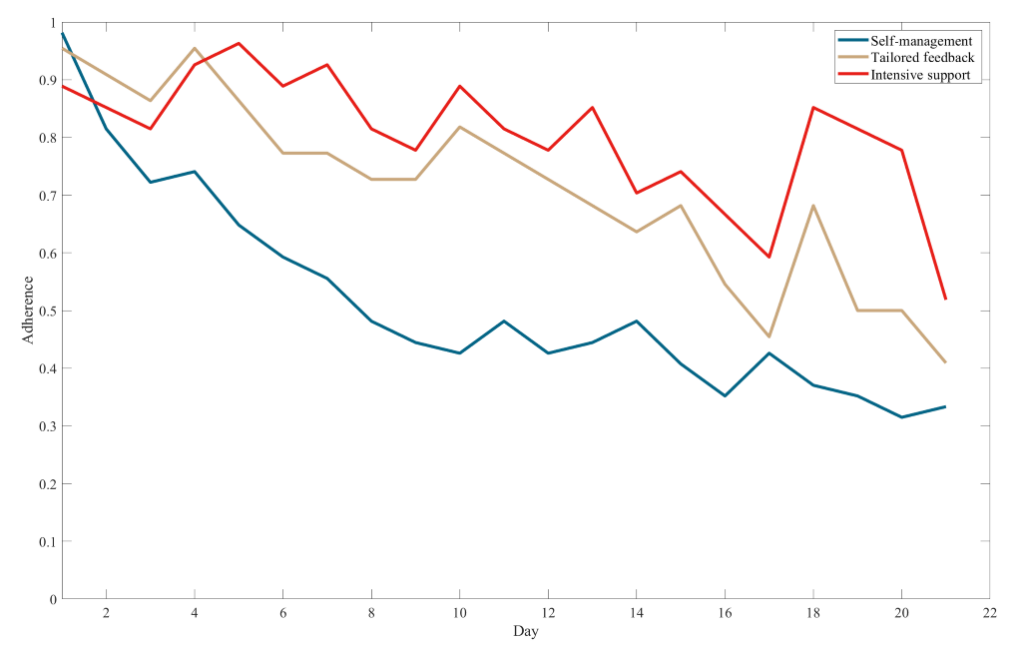
Mean values of sequences of adherence to self-monitoring of dietary behaviors for participants in each group over 21 days.

Figure 2 illustrates the sequences of adherence to self-monitoring of dietary behaviors in the 3 groups. The horizontal axis represents time in days, and the vertical axis displays the average adherence to self-monitoring of dietary behaviors, normalized by the sample size.

**Figure 3:**
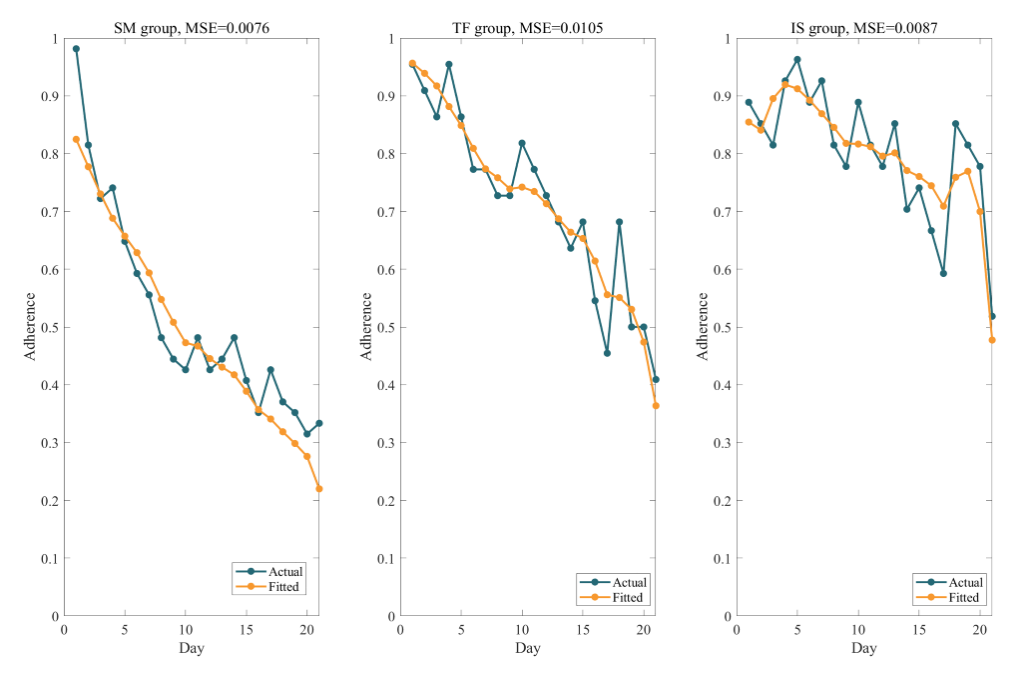
Fitted versus actual curves for each group of sequence means of adherence to self-monitoring of dietary behaviors. IS: intensive support; MSE: mean square error; SM: self-management; TF: tailored feedback.

Model coefficients and evaluation metrics are summarized in Table 4, and Figure 3 depicts the comparison between predicted and actual values. The MSE values for all 3 models approached zero, and the RMSE values remained below 0.1. These findings suggest that the ACT-R model effectively captured the participants’ patterns of adherence to self-monitoring of dietary behaviors. However, the goodness of fit for the intensive support group was comparatively low, suggesting that unaccounted factors may have influenced the model specific to the intensive support group.

**Figure 4:**
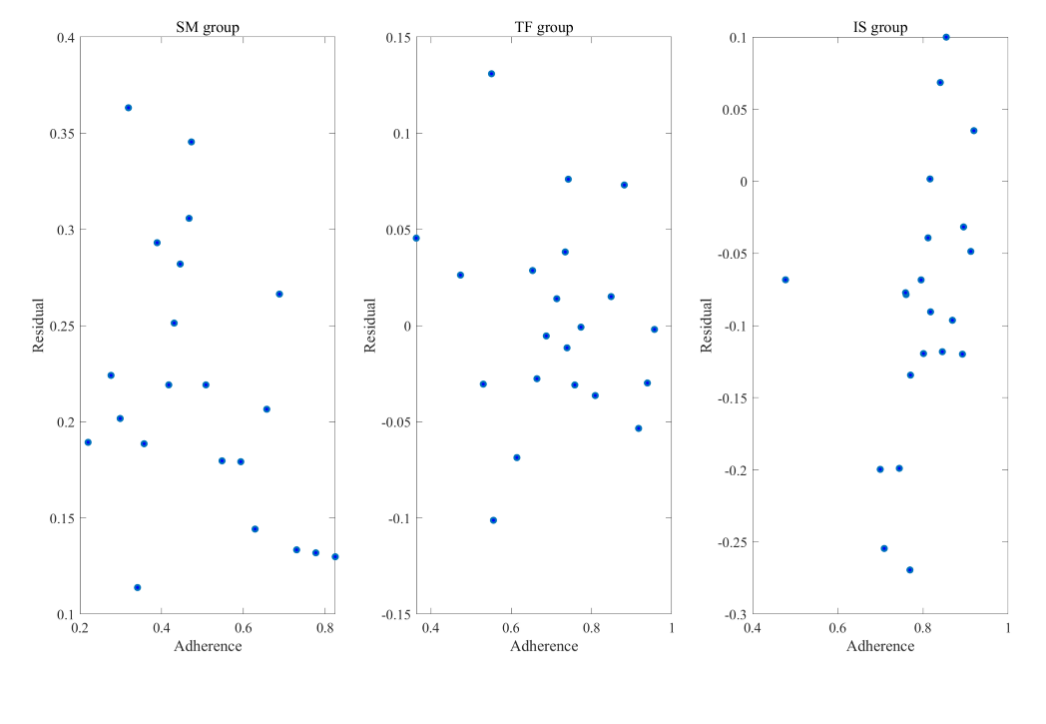
Scatter plots of models for each intervention group. IS: intensive support; SM: self-management; TF: tailored feedback.

The residual plot in Figure 4 demonstrates a random distribution of residuals, with no discernible patterns, supporting the adequacy of the model fit. The Ljung-Box test revealed no significant auto-correlation in the residuals for the self-management and tailored feedback groups. However, significant auto-correlation was detected for the intensive support group (*P*<.001), suggesting the presence of uncaptured dynamics in their adherence patterns.

**Table 4:** Model coefficients and evaluation results.

Variable		Self-management group	Tailored feedback group	Intensive support group
**Parameter, value**				
	β_0_	1.3245	3.2422	3.6510
	β_1_	–0.6909	–1.6991	–2.2174
	Tr	1.8575	3.6615	3.1929
	U_0_	0.1959	1.9833	–0.3126
	R_0_	0.7983	1.9909	4.7796
**Evaluation indicator, value (90% CI)**				
	MSE^a^	0.0104 (0.0004 to 0.0201)	0.0076 (0.0036 to 0.0157)	0.0087 (0.0040 to 0.0159)
	RMSE^b^	0.0990 (0.0660 to 0.1418)	0.0844 (0.0602 to 0.1254)	0.0911 (0.0632 to 0.1263)
	R^2^	0.6005 (0.1020 to 0.8404)	0.7339 (0.3601 to 0.8961)	0.3958 (–0.1711 to 0.7102)
**Ljung-Box test**				
	Value	11.8602	19.1268	56.2582
	*P*	.92	.51	<.001

^a^MSE: mean square error.

^b^RMSE: root mean square error.

**Figure 5:**
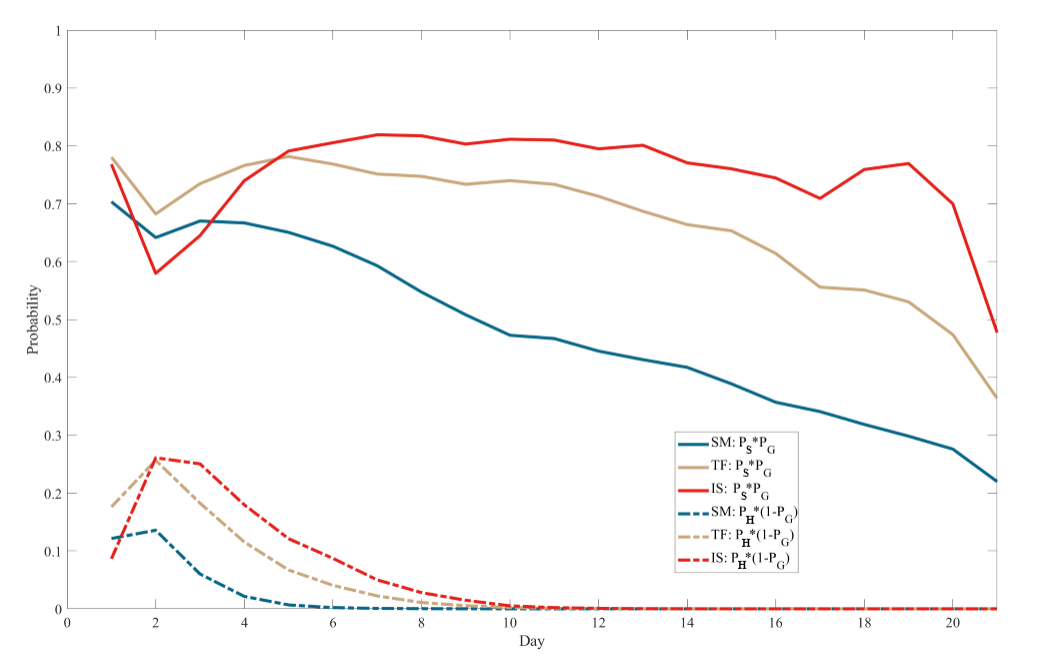
Probability curves for contributions corresponding to different mechanisms. IS: intensive support; PG: probability of retrieving the self-monitoring of dietary behaviors goal chunk; PH: probability of successful self-monitoring of dietary behaviors execution based on the habit formation mechanism; PS: probability of successful self-monitoring of dietary behaviors execution based on the goal pursuit mechanism; SM: self-management; TF: tailored feedback.

As shown in Figure 5, the model was used to illustrate the probability curves comprising 2 parts of *P_SMDB_*. Solid lines represent the contribution of goal pursuit mechanisms (*P_G_*(*n*)×*P_S_*(*n*)), while dashed lines denote the contribution of habit formation mechanisms ((1–*P_G_* (*n*))×*P_H_* (*n*)). The results indicated that the goal pursuit mechanism exhibited dominance throughout the 21-day intervention period. In contrast, the influence of the habit formation mechanism was significant only in the early stages and gradually diminished, approaching zero in the later stages. Moreover, higher levels of social support and the presence of customized feedback corresponded to higher positions for both types of curves.

## Discussion

### Predictive Modeling of Dietary Self-Monitoring in Digital Weight Loss Programs

This study presents a model developed within the framework of the ACT-R cognitive architecture, which is aimed at modeling the dynamics of adherence to self-monitoring of dietary behaviors among participants in a digital behavioral weight loss program and investigating how tailored feedback and social support influence adherence.

The model developed in this study predicts the likelihood of successful execution of self-monitoring of dietary behaviors in digital behavioral weight loss programs at any given time. Evaluation results indicate that ACT-R can provide relatively accurate assessments of historical behavior. Moreover, higher levels of social support and the presence of tailored feedback may contribute to more proactive goal pursuit and more sustained behavioral repetition.

### Interpretation of the Results

Consistent with previous research [30,33], the ACT-R architecture effectively captures the dynamics of adherence to self-monitoring of dietary behaviors over time, highlighting its potential in the design of personalized and just-in-time behavioral interventions. Nonetheless, the high heterogeneity in both the research context and population poses challenges for conducting a more profound comparative analysis with previous studies. The presence of tailored feedback and the higher levels of social support are significantly associated with increased adherence to self-monitoring of dietary behaviors.

With regard to the differential contributions of the 2 mechanisms, the goal pursuit mechanism showed a significant contribution throughout the intervention, whereas the habit-forming mechanism contributed only in the early stage of the intervention. Although participants’ social cognitive states were not collected for an in-depth analysis, this paper contextualizes several hypotheses about potential underlying causes. First, the process of recording dietary behavior might gradually be perceived as cumbersome, potentially disrupting the habit formation process. According to the cognitive load theory [50], repetitive behaviors may elevate an individual’s cognitive load and mental fatigue, ultimately resulting in decreased adherence. Second, according to the automaticity theory [51], the development of automated behaviors requires sustained repetition and reinforcement over an extended period. Thus, a short intervention duration may limit the influence of the habit formation mechanism, indicating that most participants were likely still in the early stages of behavior change and had not yet fully transitioned to forming stable habits. Third, based on the health belief model [52] and expectancy-value theory [53], behavior motivation is influenced by expected benefits. Participants’ initial adherence to self-monitoring of dietary behaviors, driven by novelty and incentives (eg, tailored feedback), was likely to diminish over time as perceived benefits declined. It may weaken motivation, thereby impeding the consolidation of habits. Fourth, according to dietitians in a past implementation evaluation study [37], tailored feedback became increasingly repetitive and monotonous later in the intervention, especially for participants struggling with hard-to-change poor dietary habits, such as those influenced by environmental constraints or social pressures. Habit formation may fail when feedback fails to adapt to participants’ needs. In summary, perceived burden and repetitiveness of self-monitoring of dietary behaviors on a phone, limited duration of interventions, declining participant motivation, and monotonous or less adaptive feedback may collectively weaken the contributions of habit formation and both mechanisms to sustained behavior change.

Notably, the applicability of the model is confined to three key aspects: (1) it is specifically designed for time-event datasets representing routine health behaviors with consistent intervals and does not apply to sporadic or irregular unhealthy behavior events; (2) it targets the analysis of high-frequency habits performed at least once daily; and (3) it requires a clearly defined starting point for behavior repetition. In this study, the starting point was set as the first day following a 7-day preparation phase, during which participants developed the necessary skills for self-monitoring of dietary behaviors. This preparation ensured a standardized baseline for habit formation analysis.

Future research can leverage the model developed in this study to explore how behavior changes under varying levels of support and feedback strategies, thereby facilitating the design of personalized interventions and enabling pre-experimental validation. Moreover, the model provides a framework for examining the sociocognitive states associated with specific behaviors, which necessitates a foundational understanding of the health behavior theory for effective application.

### Comparison With Prior Work

Considering that a comparison of the ACT-R model’s performance with that in prior studies is impractical, this study instead focused on comparing the adherence rates. Previous studies on self-monitoring of behavior reported adherence rates ranging from 45% to 58% on average [54]. Specifically, a systematic review noted that while many studies achieved a rate around 50%, few reached up to 75% [36]. In comparison, participants in the HDLC program exhibited better adherence to self-monitoring of dietary behaviors. For instance, the study by Payne et al [54] reported a 50% adherence rate over an 8-week period. However, similar to previous research [36,54], adherence to self-monitoring of dietary behaviors declined gradually over time in all 3 groups of the HDLC program, with the self-management group showing a notably faster decline than the tailored feedback and intensive support groups. In addition, findings from this study suggest that providing tailored feedback and emotional social support may be effective in maintaining participants’ long-term adherence to self-monitoring of dietary behaviors, and these are consistent with the findings in past studies [10,55]. A recent systematic review further supported this by showing that interventions incorporating feedback mechanisms are significantly more effective than those without these mechanisms [56].

### Limitations

There are several limitations in this study. First, the modeling was based on a small sample size and uneven sample size distribution among groups, which constrained the generalizability of the model’s performance. Furthermore, as the modeling was based on a limited observation period of 21 days, it may not fully capture the dynamic behavioral changes over a longer duration. Future studies could extend the modeling period to 8 weeks, 12 weeks, or even longer to better understand the long-term dynamics of user adherence.

Second, participant grouping based on BMI, which was intended for personalized intervention and resource allocation, resulted in a predominance of participants with normal weight in the self-management group. This imbalance may have introduced selection bias and confounding. Future research should consider conducting ACT-R modeling based on sample data from balanced groups, thereby mitigating such biases.

Third, this study focused on tailored feedback for dietary behaviors and examined adherence only to self-monitoring of dietary behavior, excluding other health behaviors such as exercise. Future ACT-R studies should broaden their scope to analyze additional health behaviors and their coexistence.

Fourth, the performance evaluation of the intensive support group was less satisfactory compared to that of the self-management and tailored feedback groups, likely due to potential, unexplored influencing factors. Future research should aim to optimize the ACT-R model under intensive support interventions.

Finally, no formal sample size calculation was conducted prior to analysis due to the absence of a widely accepted method for estimating the minimum sample size in ACT-R modeling. Nonetheless, the sample sizes in all 3 groups exceeded the minimum sample size recommended by the TRIPOD statement, which suggests a rule of thumb of at least 10 outcome events per parameter [41].

### Conclusion

To the best of our knowledge, this study is the first to model the adherence dynamics of self-monitoring of dietary behaviors using ACT-R under different mHealth intervention strategies. It demonstrates that this approach can quantify behavioral changes over time, providing a powerful tool for understanding compliance dynamics and refining intervention impact mechanisms. Furthermore, the study indicates that strengthening feedback with social support can effectively promote compliance, providing valuable insights for optimizing feedback strategies. Future research can explore several aspects in greater depth. First, extending the intervention duration could provide insights into the sustained effects on behavioral adherence, helping achieve a better understanding of behavioral automation and habit formation. Second, incorporating social cognitive factors could shed light on the intrinsic mechanisms of behavioral compliance. Third, leveraging this model as a foundation for dynamic algorithms could drive the development of automated intervention techniques, enabling the design of effective just-in-time adaptive interventions.

## Appendix

Multimedia Appendix 1 TRIPOD+AI checklist.

Multimedia Appendix 2 Descriptions of interventions for the 3 groups in the Health Diary Lifestyle Change program.

Multimedia Appendix 3 Intervention outcomes measured at baseline and 28-day follow-up.

## Abbreviations

ACT-R: Adaptive Control of Thought-Rational
HDLC: Health Diary for Lifestyle Change
MSE: mean square error
RMSE: root mean square error

## Data Availability

The datasets and code used in this study are available from the corresponding author upon reasonable request.
