# Supplementary material for: Exploring the Dynamics of Dietary Self-Monitoring Adherence Among Participants in a Digital Behavioral Weight Loss Program: Model Development Study
Source: J Med Internet Res. 2025 Apr 25;27:e65431. doi: 10.2196/65431 (PMC12064973; doi:10.2196/65431)
Supplement: Multimedia Appendix 2 [file jmir_v27i1e65431_app2.pdf]

Multimedia Appendix 2. Descriptions of interventions for the 3 groups in the Health Diary Lifestyle Change program.

Component	Description	SM	TF	IS
Motivation in the beginning	After the baseline assessment, all participants set goal weights with dietitians and signed a commitment to lose weight. They were informed that successful weight loss can result in a bonus.	√	√	√
Sharing	Participants in the same team were encouraged to share their daily meals and progresses within a discussion group.	×	×	√
Online health education	The series of online courses "Application of Nutrition and Health Care" are delivered weekly. The 28-day health topics included nutrition, lifestyle, psychology, and exercise. Everyday health education was approached as followed: 8:00 am: 1-min voice broadcast, illustrated knowledge points; 12:00 pm (noon): popular science article.	√	√	√
Reminder	Anti-sedentary reminder at 10:00 am, water-drinking reminder at 4:00 pm are in the daily schedule executed by health manager.	√	√	√
Diet	In the team group, participants were asked to log their meals and share their food pictures. Dieticians have to summarize common unhealthy eating habits daily on WeChat.	√	√	√
	Food photos are commented on by IS dietitians with personal food advice. Reminder will be sent by health assistant if the participant forgets to upload their diet and weight.	×	√	√
Sport	Participants are advised to exercise for 150 minutes per week. A sport coach assigns a 5-minute indoor exercise task every day and offers consulting services. The coach would broadcast live video online twice a week to guide participants through indoor exercises.	√	√	√
Weight	Every week, the health assistant would announce the WeChat group members who had lost the most weight.	√	√	√

SM: self-management; TF: tailored feedback; IS: intensive support.
