# Supplementary material for: Exploring the Dynamics of Dietary Self-Monitoring Adherence Among Participants in a Digital Behavioral Weight Loss Program: Model Development Study
Source: J Med Internet Res. 2025 Apr 25;27:e65431. doi: 10.2196/65431 (PMC12064973; doi:10.2196/65431)
Supplement: Multimedia Appendix 3 [file jmir_v27i1e65431_app3.pdf]

Multimedia Appendix 3. Intervention outcomes measured at baseline and 28-day follow-up.

Group	TF	IS	Intergroup Difference
**Weight (SD), kg**			
Baseline	73.58(17.22)	68.86(12.80)	
28-day follow-up	73.05(16.72)	67.38(12.81)	
Intragroup Difference	t_{14}=4.00, P=.001	t_{19}=5.39, P<.001	t_{33}=0.80, P=.43
**BMI (SD), kg/m2**			
Baseline	27.31(5.24)	25.67(3.45)	
28-day follow-up	26.62(5.02)	25.11(3.42)	
Intragroup Difference	t_{14}=4.03, P=.001	t_{19}=5.39, P<.001	t_{38}=0.70, P=.49
**Waist circumference (SD), cm**			
Baseline	87.59(13.77)	83.56(8.88)	
28-day follow-up	85.51(13.83)	81.41(9.71)	
Intragroup Difference	t_{14}=2.07, P=.06	t_{19}=5.39, P<.001	t_{32}=-0.05, P=.96
**Body fat (SD), %**			
Baseline	36.25(5.62)	32.52(5.92)	
28-day follow-up	33.50(7.86)	32.02(6.74)	
Intragroup Difference	t_{14}=2.30, P=.04	t_{19}=5.39, P<.001	t_{32}=1.98, P=.06
